# Diagnostic and Therapeutic Challenges in Pediatric Cutaneous Melanoma: Two Case Reports From the Moroccan Population

**DOI:** 10.7759/cureus.60999

**Published:** 2024-05-24

**Authors:** Badr Rouijel, Yacine Zouirech, Hajar El Agouri, Nawfal Fejjal

**Affiliations:** 1 Pediatric Plastic Surgery Unit, Faculty of Medicine and Pharmacy of Rabat, Children's Hospital of Rabat, Mohamed V University of Rabat, Rabat, MAR; 2 Department of Pathology, Pediatric Plastic Surgery Unit, Faculty of Medicine and Pharmacy of Rabat, Mohammed V Military Hospital Rabat, Mohammed V University of Rabat, Rabat, MAR

**Keywords:** surgical oncology, plastic reconstruction, excision, children, cutaneous melanoma

## Abstract

Although cutaneous melanoma (CM) is one of the most prevalent cancers in adults, it is rarely reported in children. Often, the diagnosis is delayed and difficult to make. We presented two novel examples of pediatric CM from the Department of Pediatric Plastic Surgery Unit at Rabat Children's Hospital. The first case included a 14-year-old girl who had a cutaneous nodule on her right leg. She first came with an inguinal enlargement, for which a lymph node biopsy was positive. A further inguinal dissection of 10 lymph nodes revealed four metastatic ones (4N+\10N). She received a wide local excision of the lesion, which revealed nodular melanoma with an 8 mm thickness as determined by Breslow, as well as safe lateral and deep margins. The course was distinguished by the emergence of new metastatic lymph node locations, and the patient died a few weeks later. The second case included a 13-year-old girl who appeared with a cutaneous lesion centered on a scar on her right leg. She also underwent a large local excision, which revealed nodular melanoma with a thickness of 12 mm according to Breslow, as well as complete lateral and deep excisions. Her follow-up revealed favorable results, with no local recurrence or distant metastases. This case series emphasized the difficult management of two separate occurrences of pediatric CM. We also emphasized the importance of early detection of suspicious lesions, regular follow-ups, and raising awareness among high-risk patients.

## Introduction

Cutaneous melanoma (CM) is a highly malignant tumor caused by the degeneration of pigment-producing cells called melanocytes. The occurrence of CM is increasing worldwide. This entity is rare in infancy and adolescence (age < 20 years), accounting for around 1% to 4% of all melanomas and 3% to 4% of all pediatric cancers [1]. Many underlying conditions enhance the likelihood of developing CM in children (e.g., enormous congenital melanocytic nevi, familial dysplastic nevus syndrome, and xeroderma pigmentosum) [2].

CM is frequently diagnosed late due to a lack of suspicion and clinical-histological similarities with other diseases. Furthermore, clinical care of CM is typically difficult and suffers from a lack of knowledge and clinical competence [3]. Although pediatric CM presents differently from adult CM, there are no particular management guidelines for children.

## Case presentation

Case 1

**Figure 1:**
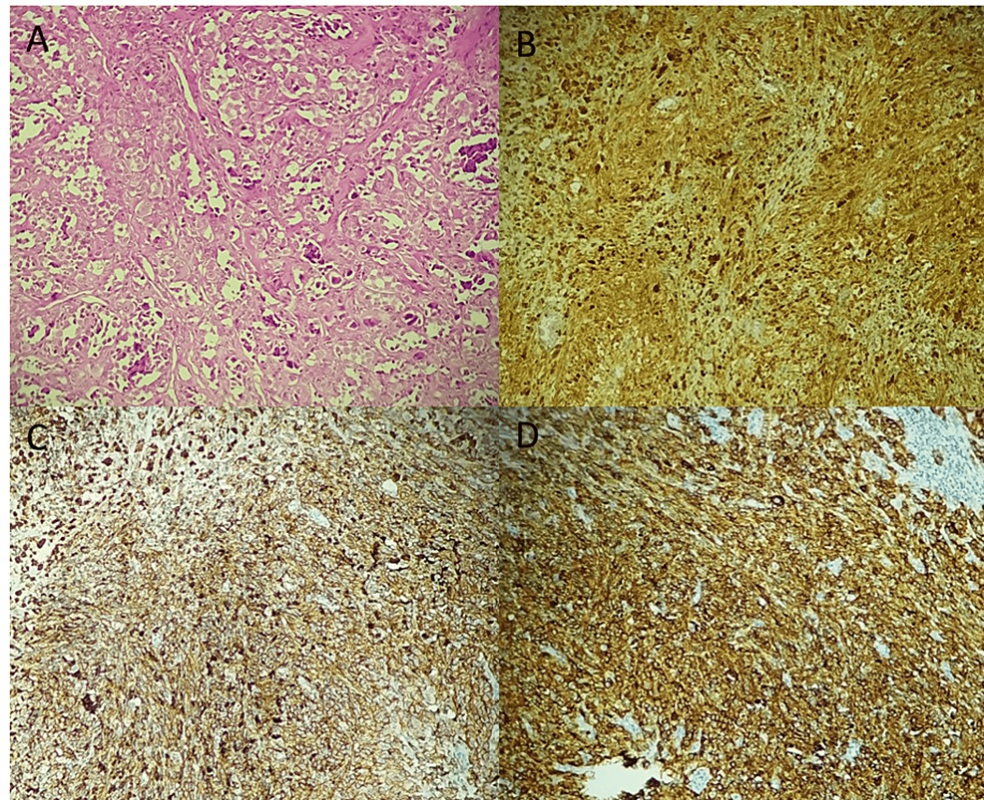
Microscopic findings showing massively infiltrated lymph node tissue by malignant tumor proliferation with large cells, irregular hyperchromatic nuclei, sometimes monstrous and highly nucleolated: (A) hematoxylin stain, x100. Tumor cells showed positive staining with anti-PS100 (B:immunostain, x100), and strong, diffuse staining with anti-HMB45 (C: immunostain, x100), and anti-Melan A antibodies (D: immunostain, x100).

The first patient was a 14-year-old, referred for treatment to our department in July 2013. She presented five months ago with a painless right inguinal swelling. The patient complained of no other symptoms, had no particular personal history, and had no family history of cancer. An excision biopsy of the inguinal lymph node was performed. Clinical examination revealed a painless tumor on the right leg, measuring approximately 1 cm in size. Also, a scar from right inguinal lymph node resection was reported. Examination of peripheral lymph node areas was normal. The rest of the physical examination was unremarkable. Histopathological examination of the initial resection of the inguinal lymph node showed a massively infiltrated lymph node tissue by a malignant tumor proliferation with polymorphic appearance and rich vascularity. It consisted of large cells, with abundant eosinophilic cytoplasm, irregular hyperchromatic nucleus, sometimes monstrous and highly nucleolated, with frequent and abnormal mitoses (Figure 1A). A panel of immunostains was performed, revealing positive staining with anti-PS100 and strong, diffuse staining with anti-Melan A and anti-HMB45 antibodies (Figures 1B-1D). The tumor cells were negative for anti-CKAE1/AE3 (cytokeratin AE1/AE3), epithelial membrane antigen (EMA), CD30, and leukocyte common antigen (LCA) antibodies. Therefore, the diagnosis of an inguinal lymph node metastasis from a pigmented melanoma was made.

The patient underwent thoracic-abdominal-pelvic CT and showed multiple bilateral para-aortic, mesenteric, and right inguinal lymph node metastasis. Meanwhile, the brain CT scan showed no evidence of brain metastasis.

**Figure 2:**
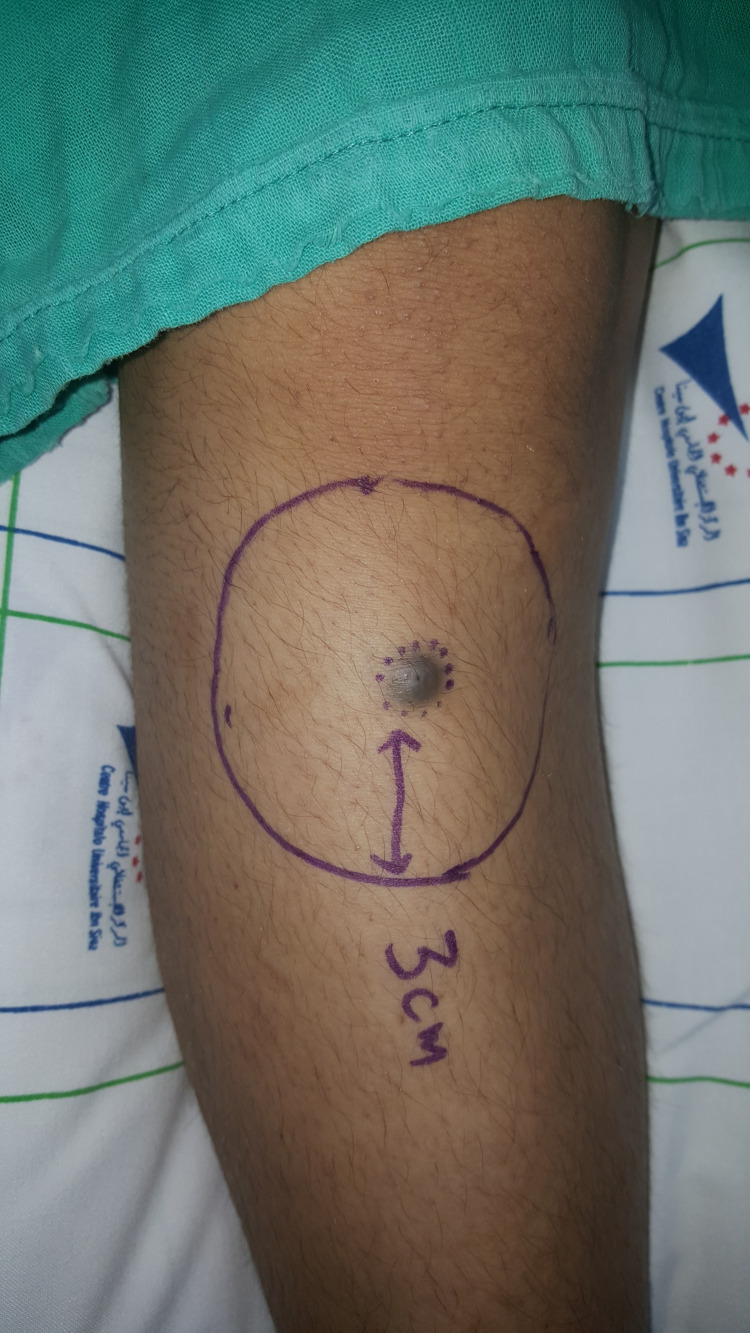
Preoperative picture of the first patient showing a 3 cm resection margin.

**Figure 3:**
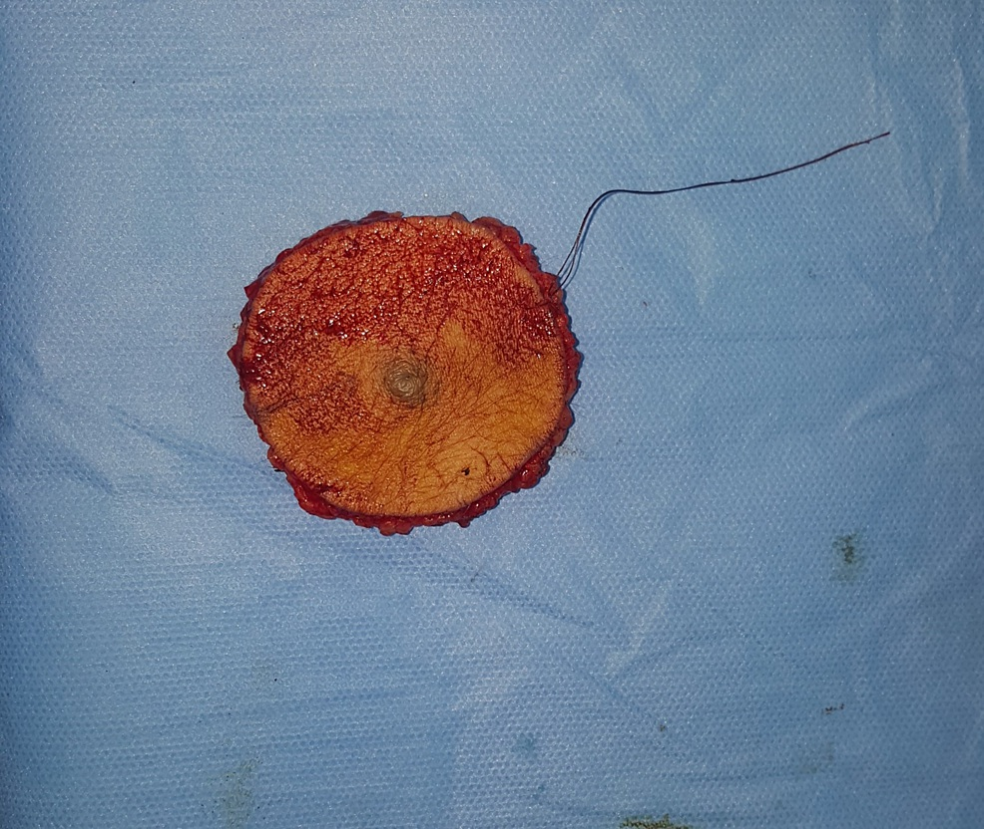
Postoperative picture of the first patient showing the surgical specimen.

**Figure 4:**
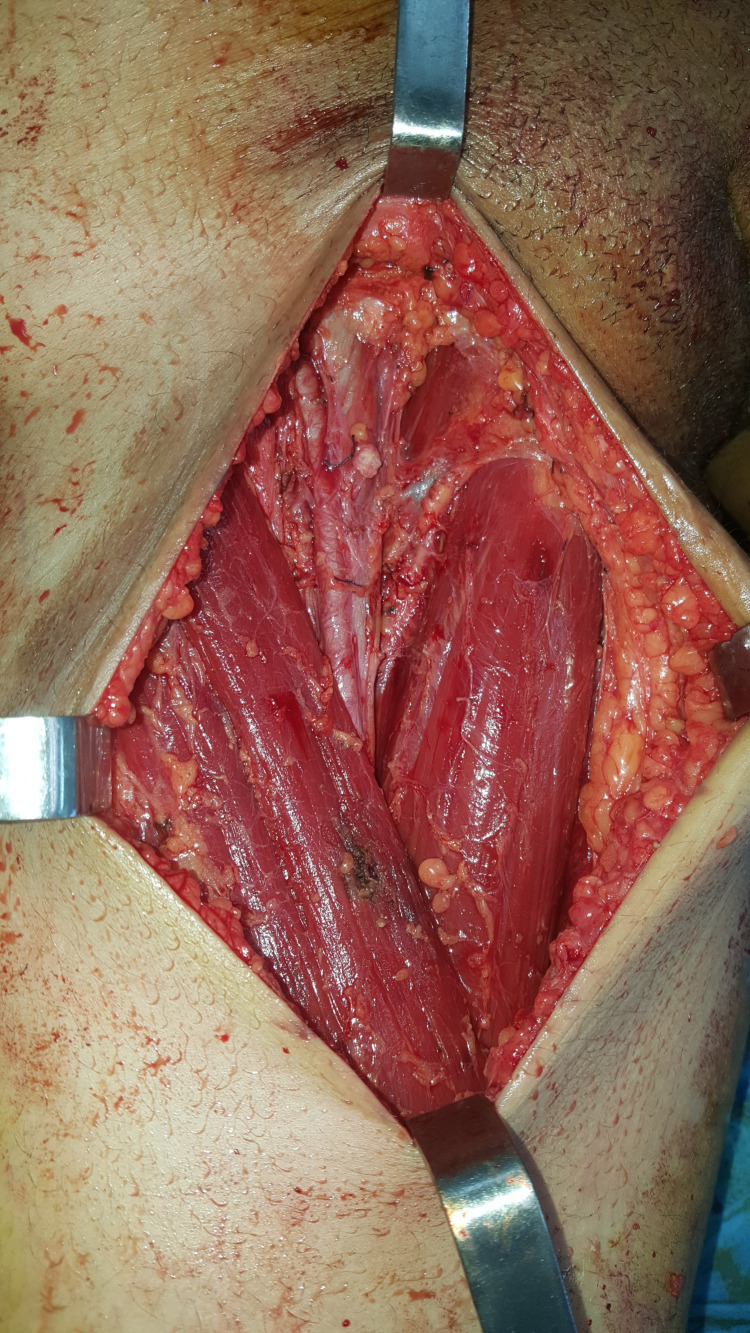
Perioperative picture of the first patient showing right inguinal lymph node dissection.

Based on these findings and following the guidelines of the American Joint Committee on Cancer (AJCC), we performed a wide local excision of the tumor with a 3 cm margin (Figures 2-3) and used a split‑thickness skin graft to cover the surgical defect. She also underwent a right inguinal lymph node dissection at our department (Figure 4). Microscopic examination of the surgical specimen concluded nodular melanoma without a horizontal component, with a thickness of 8 mm according to Breslow and Clark level V. No lymphoid infiltrate, vascular emboli, or perineural involvement was seen. Lateral and deep margins were safe. Additional inguinal dissection of 10 lymph nodes showed four metastatic ones with subcapsular infiltration (4N+\10N). Thus, the tumor was staged T4aN3 following the AJCC classification (8e edition).

Her wounds healed uneventfully within two weeks, and no postoperative complications were recorded. Therefore, she was referred to the Pediatric Hematology and Oncology Service (SHOP). The patient initially received four cycles of dacarbazine-based chemotherapy (500 mg/m^2^) without tumor regression. Then two cycles of Pembrolizumab (Keytruda) immunotherapy. The course was marked by the deterioration of her general condition when she presented new metastatic lymph node sites. A few weeks later, the patient passed away.

Case 2

The second patient, a 13-year-old, was referred to our department in September 2018. The patient had no significant past medical or surgical history. Two months ago, she presented a painless skin lesion on the right leg, with no associated symptoms. Clinical examination revealed a skin lesion on the anterior surface of the right leg, measuring approximately 5 cm in size, reddish, painless, with poorly defined borders, and centered by a scar of approximately 4 cm. Examination of peripheral lymph node areas and the rest of the physical examination were normal.

**Figure 5:**
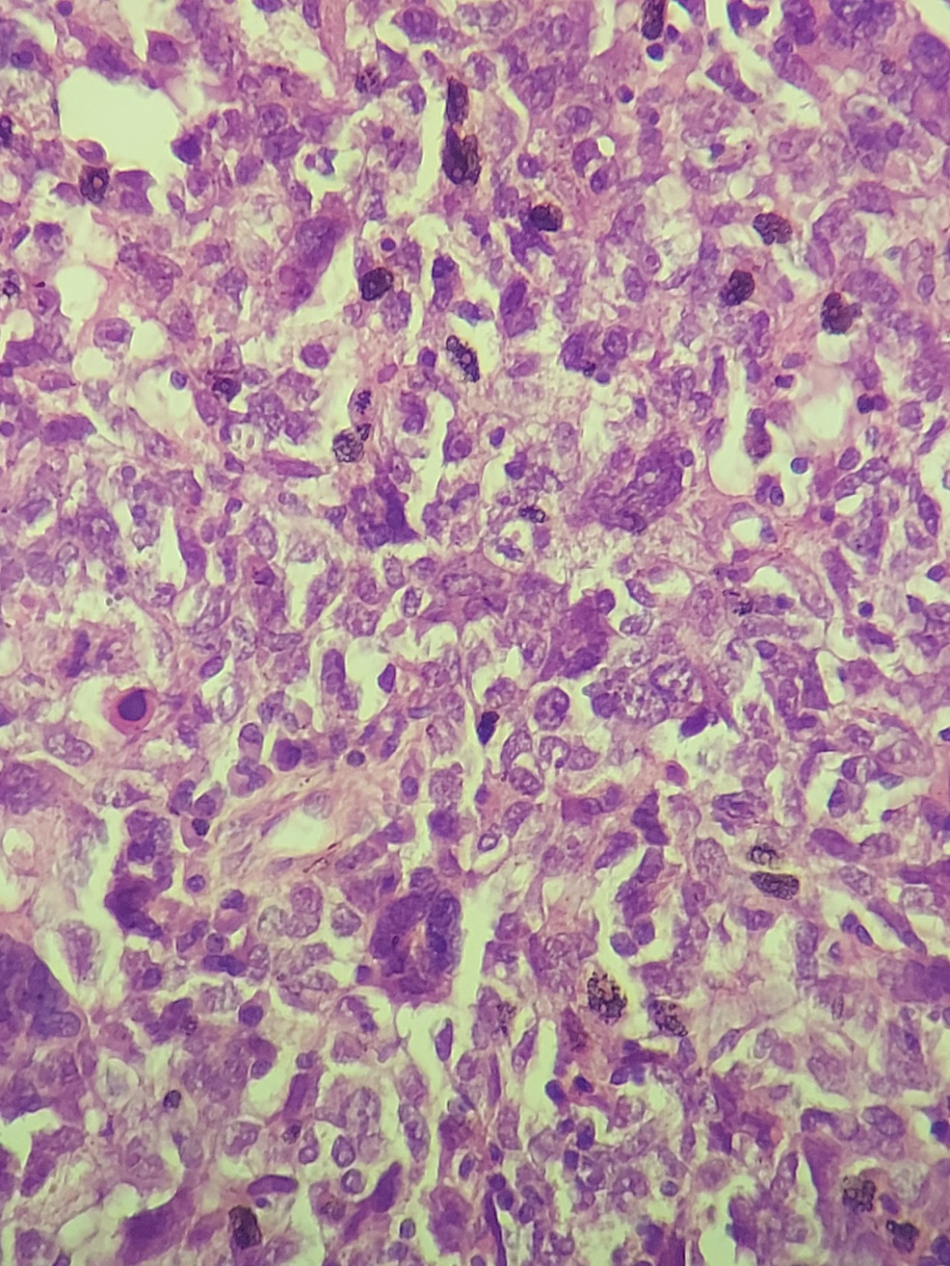
Microscopic findings showing a malignant tumor proliferation with large cells, hyperchromatic nuclei, and strong nucleoli (hematoxylin stain, x200).

The patient underwent a local excision biopsy of the lesion. Macroscopically, the excised skin piece measured 4 cm x 2 cm x 2 cm, centered by a firm and grayish-colored nodular lesion of 2 cm x 1.5 cm x 0.8 cm. Microscopically, the dermis showed a partially pigmented nodular proliferation with an intraepithelial component. Tumor cells were polygonal with hyperchromatic nuclei and strong nucleoli (Figure 5). The tumor cells were diffusely positive for anti-PS100, anti-HMB45, and anti-Melan A antibodies. Then, the diagnosis of nodular melanoma, with a thickness of 12 mm according to Breslow and Clark level V was made, with complete lateral and deep excision.

The patient underwent brain and thoracic-abdominal-pelvic CT scans. They showed no evidence of distant metastasis.

**Figure 6:**
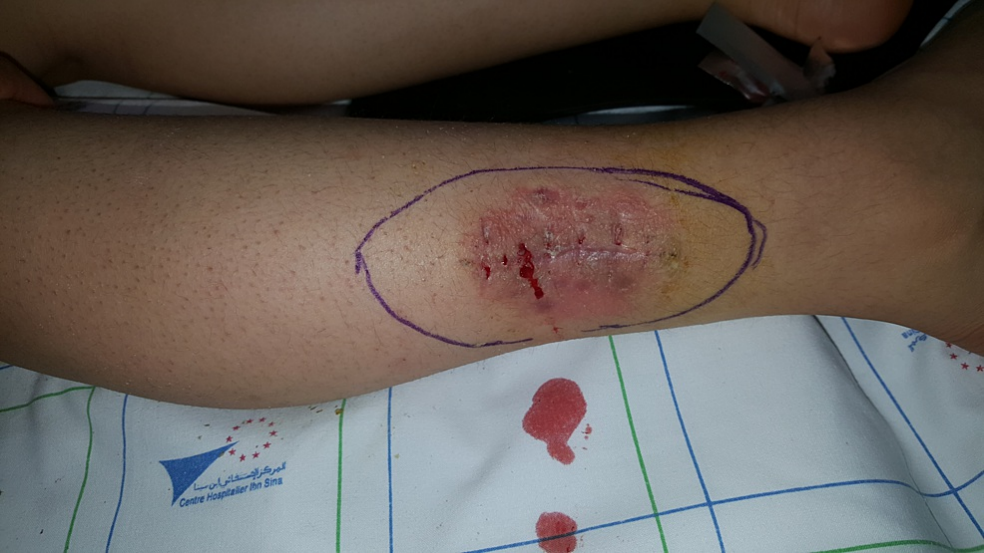
: Preoperative picture of the second patient showing a 3 cm resection margin.

Afterward, we performed a wide local excision of the tumor at a 3 cm margin with skin grafting at our department (Figure 6). Histological study of the samples revealed skin tissue bordered by partially abraded epidermis. The underlying dermis showed a dense and polymorphic inflammatory granuloma, of the resorptive type, occasionally evolving into scar tissue. No suspicious lesions were found. Then, the diagnosis was subacute resorptive inflammatory changes without visible residual tumor.

**Figure 7:**
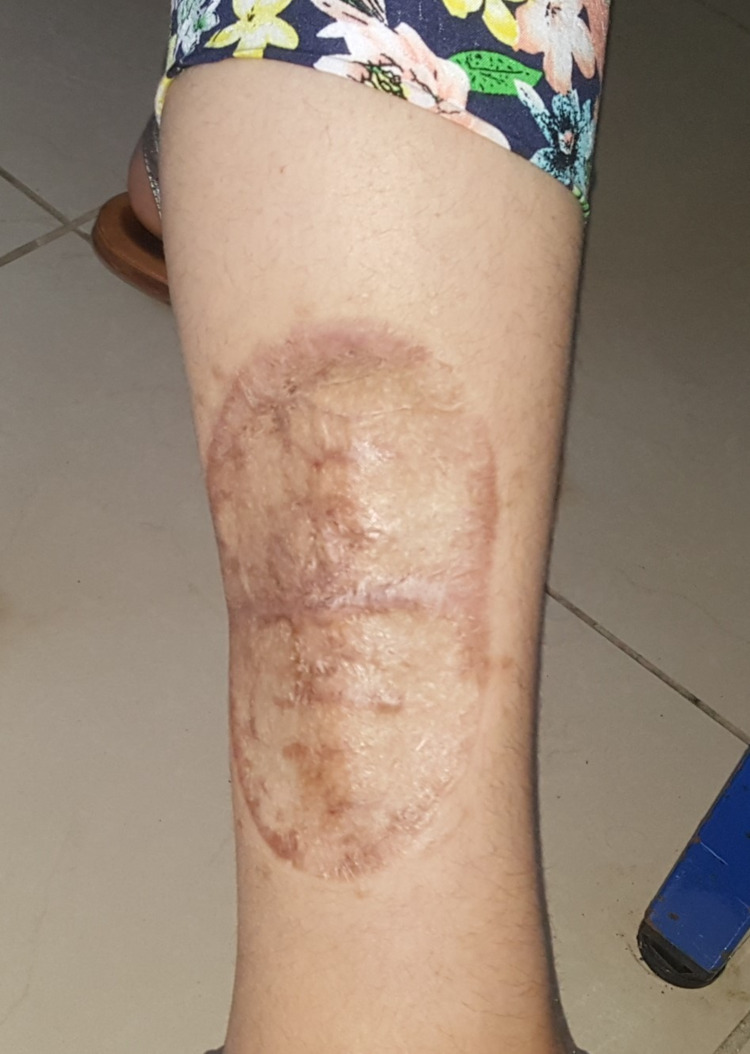
Picture of the second patient showing the scar plaque six months later after surgical excision and skin graft.

Postoperative recovery was uneventful. A follow-up, including clinical examination, and inguinal and abdominopelvic ultrasound were performed every three months for one year, then every six months for two years, then annually for five years (Figure 7). Over a period of five years, clinical and radiological follow-up showed favorable outcomes with no abnormalities, local recurrence, or distant metastasis.

## Discussion

CM is an uncommon skin cancer that is considered potentially lethal. According to a cancer statistics assessment based on a recent study conducted by the Surveillance, Epidemiology, and End Results (SEER), the rate of pediatric CM rises with age, from 4% in patients aged 0-4 years to 72% in patients aged 15-19 years [4].

According to the European Cooperative Study Group for Pediatric Rare Tumors (EXPeRT) definition, pediatric CM should be considered a very rare tumor (VRT) in children under the age of 15 (annual incidence 1.3-1.6 per million), whereas it is not in adolescents (prevalence around 15 per million in the population aged 15-19 years) [5]. Due to the higher frequency reported after puberty (most likely due to higher cumulative exposure to sunlight), the yearly rate of CM is 4.5 per million population aged 0 to 19 years [6].

When it occurs in children and teenagers, CM is known to be slightly more common in girls than boys [7], as shown in our study. Furthermore, it has been noted that the anatomic distribution varies with age; primary CM is more commonly found on the head and neck of youngsters and occasionally on the trunk as compared to teenagers and young adults [8].

Although sharing similar risk factors with adult CM (e.g., fair skin, sun exposure, and a history of sunburns), other studies suggest additional factors such as congenital, dysplastic, or numerous nevi, family history of melanoma, genetic disorders like xeroderma pigmentosum, and acquired or congenital immunosuppression [9]. In our paper, we found that neither patient had any specific risk factors.

Clinical care of children and adolescents with CM tends to be a challenge. Pediatric ABCDE (A, amelanosis; B, bleeding/ulceration; C, uniform color; D, variable diameter; and E, evolving features) criteria have been documented in less than 40% of cases [10]. In terms of classification: melanoma developing on congenital nevi, conventional melanoma, which is clinically and molecularly comparable to adult-type melanoma, and spitzoid melanoma have all been identified as distinct subtypes [11]. The genetic profile of each of these categories varies, which is critical to determining risk factors, histopathological findings, and therapy options [12]. In our study, conventional melanoma was diagnosed but deferred due to the patient's age and unusual presentation.

Furthermore, the clinical and pathologic characteristics of CM in children frequently render diagnosis difficult because of the clinical and histological overlap with other diseases, like the spectrum of spitzoid tumors and other non-melanoma skin tumors, necessitating the involvement of experienced dermatologists and pathologists [13]. Patients frequently have amelanotic lesions of larger thickness, a greater probability of regional lymph node involvement, and a generally less aggressive clinical course [14].

Despite its diverse appearance and course, as demonstrated in our case series, there are no clear therapeutic protocols for the medical management of pediatric CM. Surgery is considered the cornerstone of treatment and first-line treatment in many cases. To avoid local recurrence, the AJCC melanoma staging committee for adults [15] recommends a thorough and wide local excision with sufficient margins based on Breslow's thickness (2-3 cm). It is critical to note that the margins should be circumferential, encompassing the previous biopsy site. After surgery, a split-thickness skin graft is necessary to cover the surgical blemish. In the case of limbs, it is important to highlight that skin grafts should always be taken from the contralateral limb to prevent confusion between lymph node metastases and graft-related infection.

The indication of sentinel lymph node biopsy (SLNB) for staging pediatric melanoma is currently under debate. However, in the case of regional involvement, total lymph node dissection is still being debated due to the increased risk of complications [16-19]. In the first patient, an inguinal lymph node biopsy was initially done and later followed by an inguinal lymph node dissection at our department.

Management of locally advanced and metastatic stages includes chemotherapy, immunotherapy, or targeted therapy. The US Food and Drug Administration has recently updated the indications for several immunotherapy agents to cover the treatment of unresectable or metastatic pediatric CM [20]. In this study, both patients did not behave in the same manner, yet they received the necessary treatment. Indeed, the first patient was diagnosed in the metastatic phase. We decided to employ immunotherapy and conventional antimitotics (Dacarbazine) with meticulous monitoring.

In addition, it has been observed that most recurrences and melanoma-related mortality occur in pediatric melanoma patients more than five years after first diagnosis [12]. As a result, long-term follow-up is critical for pediatric patients. There are currently no standard recommendations for follow-up investigations in cases of pediatric CM.

The medical management of pediatric CM remains difficult. According to the EXPeRT [15], the most significant proposals are to examine pediatric cases in multidisciplinary teams that include both pediatric and adult physicians. In addition, young patients must be enrolled in prospective trials. Furthermore, an effective worldwide collaboration between pediatric and adult melanoma entities is needed to ease the transfer of potentially beneficial novel treatments from the adult to the pediatric setting.

## Conclusions

Despite its rarity, pediatric CM remains a public health problem. The diagnosis is typically delayed, and patients are frequently presented with advanced stages. To our knowledge, the patients that we were reporting are unique cases within the Moroccan population published in the literature thus far. Nevertheless, given the absence of a national registry, there isn't any recorded data on childhood melanomas.
